# Supplementary material for: A FRET biosensor reveals spatiotemporal activation and functions of aurora kinase A in living cells
Source: Nat Commun. 2016 Sep 14;7:12674. doi: 10.1038/ncomms12674 (PMC5027284; doi:10.1038/ncomms12674)
Supplement: Supplementary Information — Supplementary Figures 1-5 [file ncomms12674-s1.pdf]

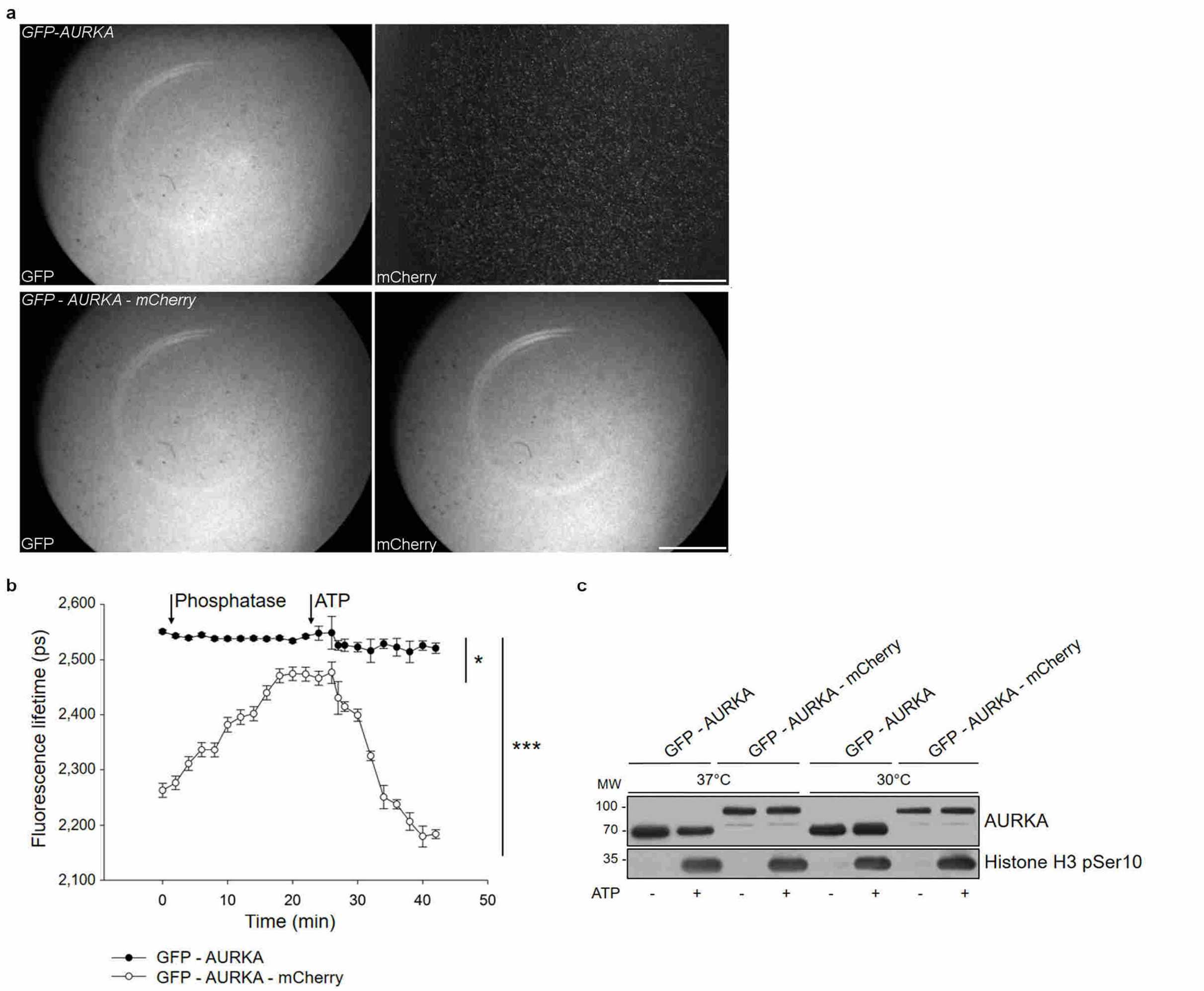

Supplementary Figure 1 | The AURKA biosensor is autophosphorylated and it is catalytically active at 30°C and at 37°C. (**a**) Representative fluorescence micrographs of the donor and the acceptor fluorophores for the GFP-AURKA and the GFP-AURKA-mCherry purified proteins. Scale bar: 5 µm. (**b**) Quantification of EGFP lifetime images taken every 2 min from GFP-AURKA and GFP-AURKA-mCherry samples incubated at 37°C and treated with λPP for 25 min and then with ATP for 20 min. Data represent means ± s.e.m. of three independent experiments. (**c**) Representative *in vitro* kinase assay and western blot analysis showing the abundance of a Ser10-phosphorylated band of histone H3 in samples containing GFP-AURKA or GFP-AURKA-mCherry and incubated with ATP at 30°C for 1 hour or at 37°C for 30 min. Loading control: total AURKA. ****P*<0.001 against each timepoint in the corresponding "GFP-AURKA" condition; ns: not significant. Statistical test: Two-way ANOVA.

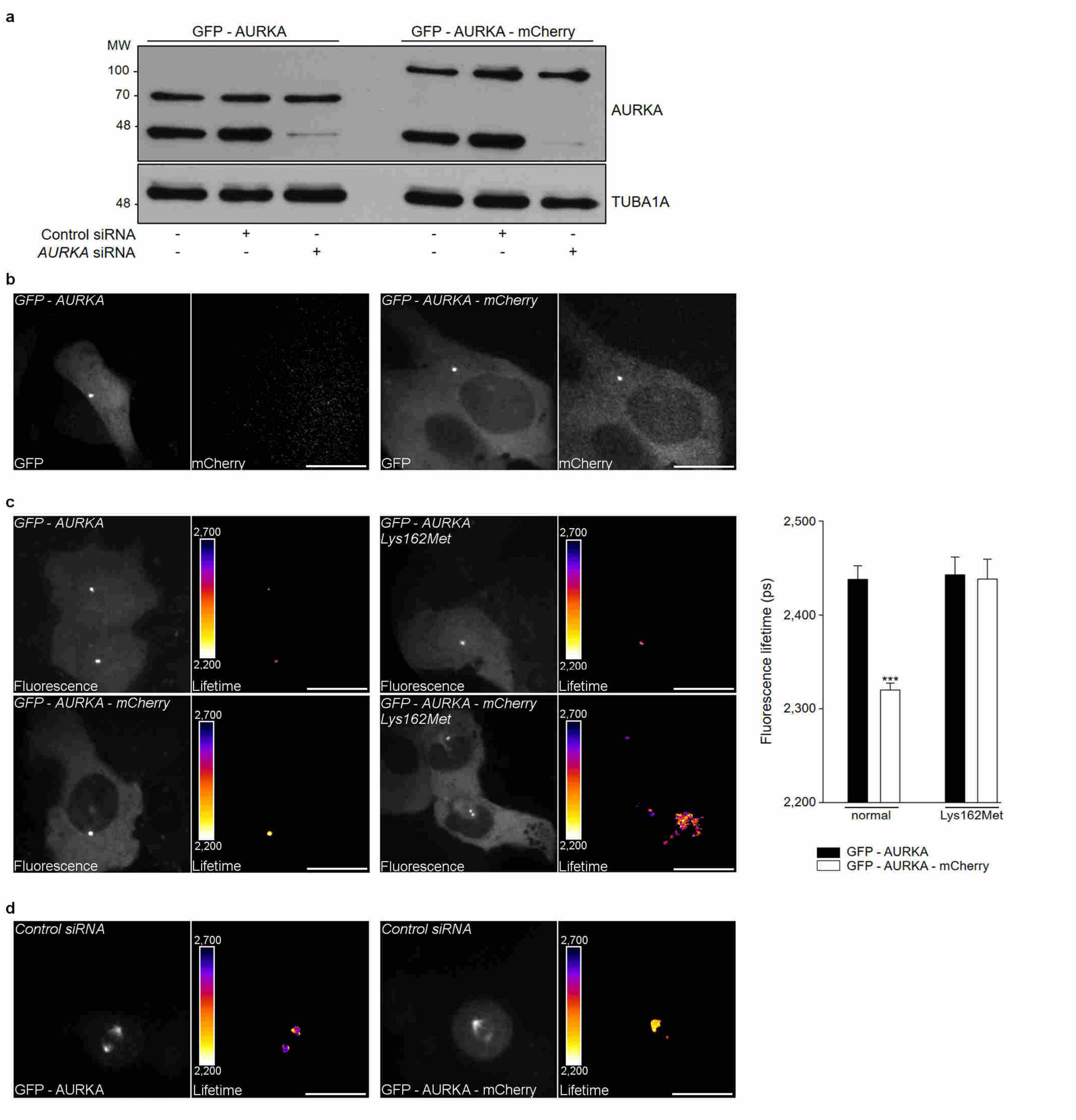

Supplementary Figure 2 | The Lys162Met variant of the AURKA biosensor is inactive *in cellulo*. (**a**) Representative western blot illustrating the efficacy of the downregulation strategy for endogenous *AURKA* in GFP-AURKA and in GFP-AURKA-mCherry cells. Loading control: TUBA1A. (**b**) Representative fluorescence micrographs of the donor and the acceptor fluorophores for the GFP-AURKA and the GFP-AURKA-mCherry U2OS stable cell lines. (**c**) (left and middle panels) Representative fluorescence (GFP channel) and lifetime images of U2OS cells stably transfected with normal or Lys162Met GFP-AURKA or GFP-AURKA-mCherry. (right panel) Quantification of the lifetime of EGFP in centrosomes from interphase cells stably transfected as in the left and in the middle panels.

n = 10 to 15 cells in one experiment representative of three. (**d**) Representative fluorescence (GFP channel) and lifetime images of GFP-AURKA and GFP-AURKA-mCherry cells transfected with a control siRNA and synchronised at mitosis. Data are means ± s.e.m. Pseudocolor scale: pixel-by-pixel lifetime. Scale bar: 10 µm. ****P*<0.001 against the corresponding "GFP-AURKA" condition. Statistical test: One-way ANOVA.

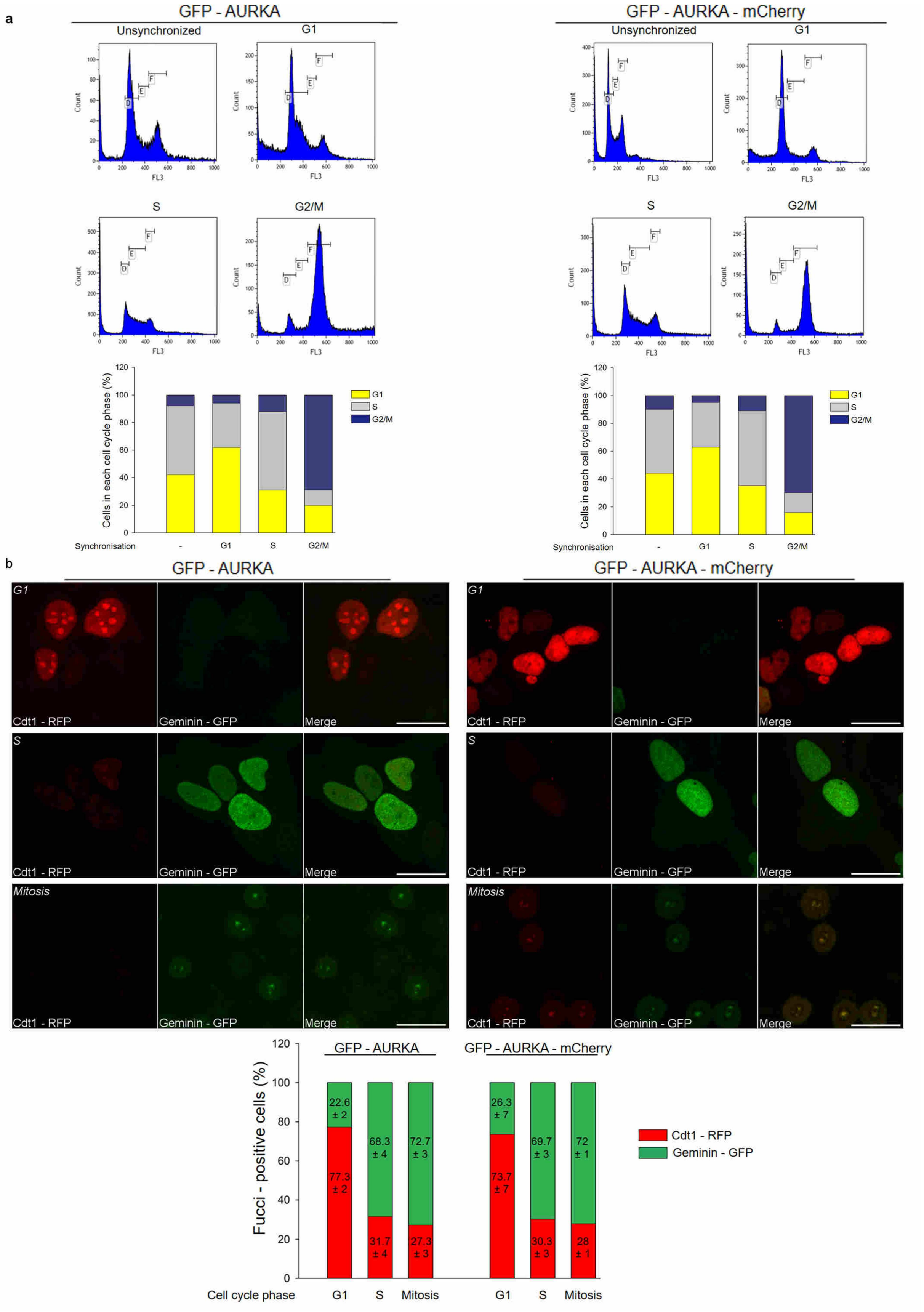
a
GFP - AURKA
Unsynchronized
G1
S
G2/M
GFP - AURKA - mCherry
Count
FL3
Cells in each cell cycle phase (%)
Synchronisation
G1
S
G2/M
b
GFP - AURKA
GFP - AURKA - mCherry
Cdt1 - RFP
Geminin - GFP
Merge
Mitosis
Fucci - positive cells (%)
Cell cycle phase
22.6 ± 2
77.3 ± 2
68.3 ± 4
31.7 ± 4
72.7 ± 3
27.3 ± 3
26.3 ± 7
73.7 ± 7
69.7 ± 3
30.3 ± 3
72 ± 1
28 ± 1

Supplementary Figure 3 | Efficiency of the cell synchronisation procedures. (**a**). FACS plots and corresponding stacked histograms illustrating the proportion of GFP-AURKA and of GFP-AURKA-mCherry cells synchronised in each cell cycle phase. D, E and F in the FACS plots indicate the populations of cells in G1, S or G2/M phase. (**b**) Representative images and corresponding stacked histograms quantifying the percentage of GFP-AURKA and of GFP-AURKA-mCherry cells synchronised as indicated and stained with the FUCCI sensor localising in the nucleus. The FUCCI technology is based on the oscillation of two fluorescently labelled DNA replication factors specific to the G1 and the S/G2/M phases, Cdt1 and Geminin. Cells were fixed in 4% PFA to preserve the fluorescence of GFP-AURKA and of GFP-AURKA-mCherry. n = 100 cells scored per conditions from one experiment representative of three. Data are means of three independent experiments. Scale bar: 10 μm.

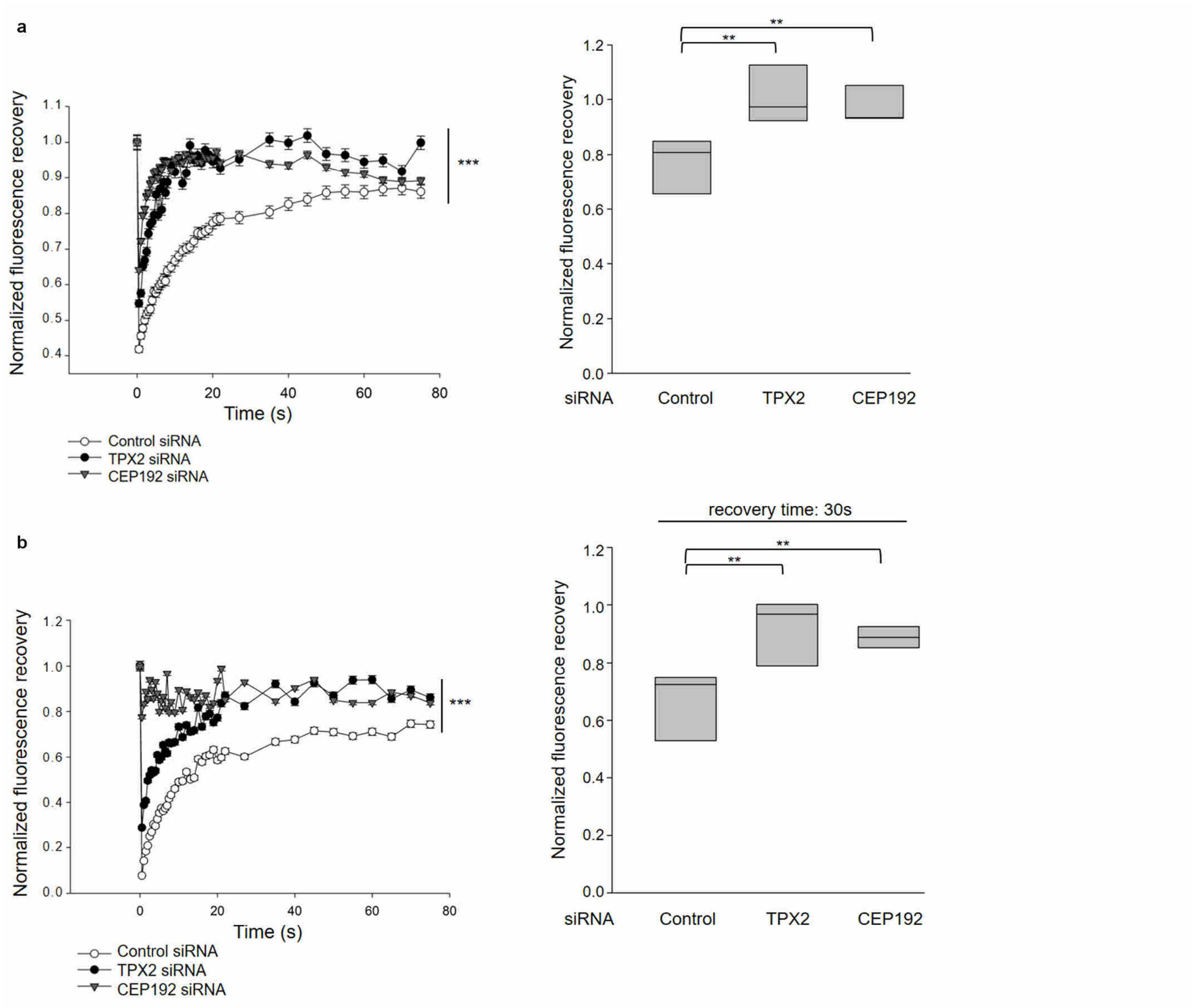

Supplementary Figure 4 | The AURKA biosensor shows intracellular motility after the depletion of *TPX2* or *CEP192* in the G1 phase or at mitosis. (left panels) Quantification of the fluorescence recovery in GFP-AURKA-mCherry cells transfected with a control, *TPX2*- or *CEP192*-specific siRNA, synchronised at mitosis (**a**) or in G1 (**b**), and subjected to FRAP analysis. n = 30 cells per condition from three independent experiments. Data represent means ± s.e.m. (right panels) Degree of fluorescence recovery after 30s from the photobleaching procedure. ***P*<0.01 and ****P*<0.001 against the corresponding "Control siRNA" condition. Statistical test: One-way ANOVA.

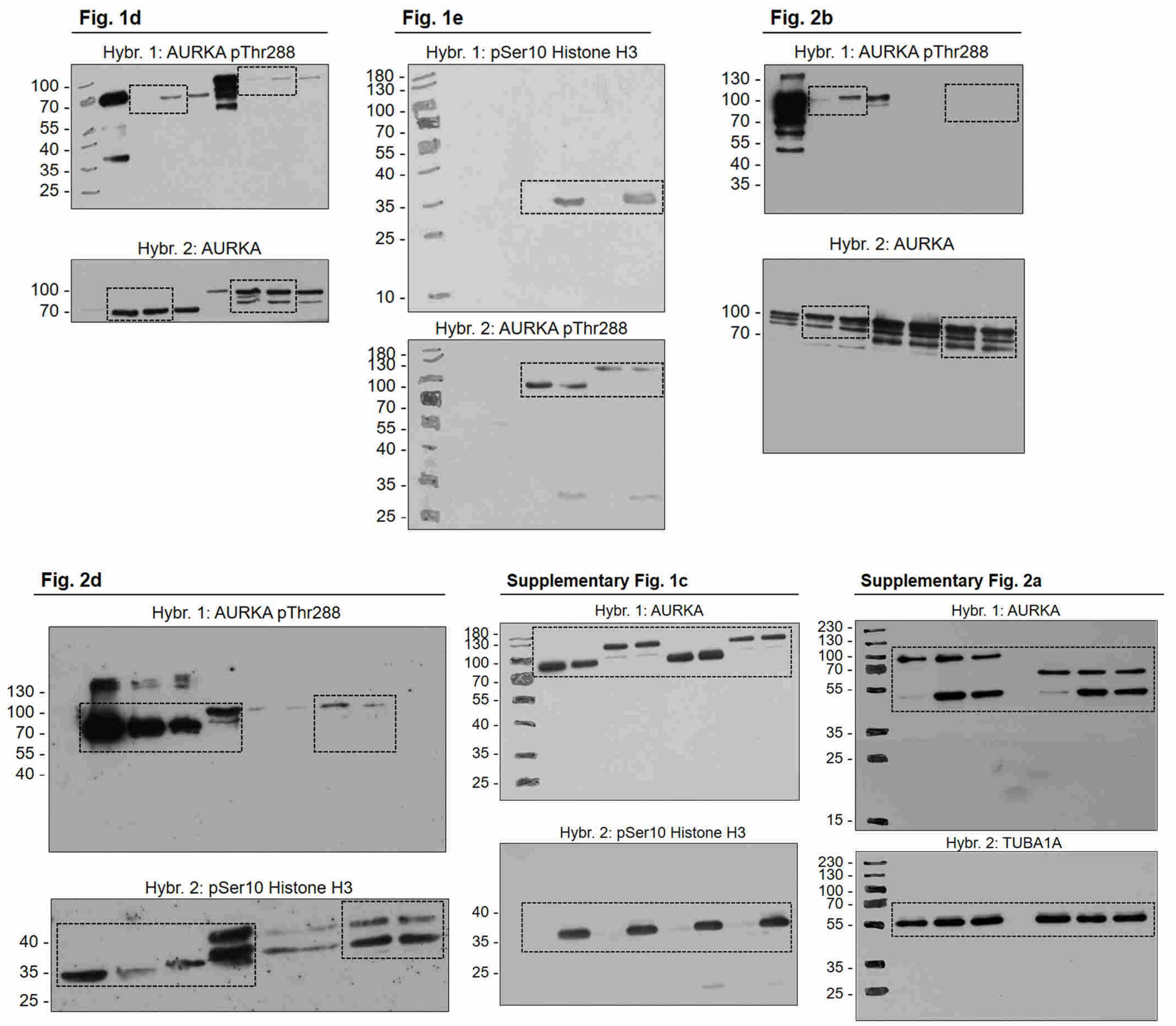

Supplementary Figure 5 | Representative uncropped blots. Scans of the full blots used in the study. The antibody used and the figure to which they correspond are indicated above each blot. The area enclosed by a dashed rectangle represents the portion of the blots integrated in the figures of the study.
